# Proteomic Investigation of the Sinulariolide-Treated Melanoma Cells A375: Effects on the Cell Apoptosis through Mitochondrial-Related Pathway and Activation of Caspase Cascade

**DOI:** 10.3390/md11072625

**Published:** 2013-07-22

**Authors:** Hsing-Hui Li, Jui-Hsin Su, Chien-Chih Chiu, Jen-Jie Lin, Zih-Yan Yang, Wen-Ing Hwang, Yu-Kuei Chen, Yu-Hsuan Lo, Yu-Jen Wu

**Affiliations:** 1 National Museum of Marine Biology and Aquarium, Pingtung 94450, Taiwan; E-Mails: hhli@nmmba.gov.tw (H.-H.L.); x2219@nmmba.gov.tw (J.-H.S.); 2 Department of Biotechnology, Kaohsiung Medical University, Kaohsiung 80761, Taiwan; E-Mail: woodnettle2002@gmail.com; 3 Graduate Institute of Veterinary Medicine, National Pingtung University of Science and Technology, Pingtung 91202, Taiwan; E-Mail: q87634@yahoo.com.tw; 4 Graduate Institute of Food Science, National Pingtung University of Science and Technology, Pingtung 91202, Taiwan; E-Mail: gini0307@yahoo.com.tw; 5 Department of Food Science and Nutrition, Meiho University, Pingtung 91202, Taiwan; E-Mails: x00000018@meiho.edu.tw (W.-I.H.); x00008396@meiho.edu.tw (Y.-K.C.); 6 Excellence Biotech Co., Kaohsiung 80655, Taiwan; E-Mail: yn1581@yahoo.com.tw

**Keywords:** melanoma, sinulariolide, proteomic, mitochondria, apoptosis, caspase cascade

## Abstract

Sinulariolide is an active compound isolated from the cultured soft coral *Sinularia flexibilis*. In this study, we investigated the effects of sinulariolide on A375 melanoma cell growth and protein expression. Sinulariolide suppressed the proliferation and migration of melanoma cells in a concentration-dependent manner and was found to induce both early and late apoptosis by flow cytometric analysis. Comparative proteomic analysis was conducted to investigate the effects of sinulariolide at the molecular level by comparison between the protein profiles of melanoma cells treated with sinulariolide and those without treatment. Two-dimensional gel electrophoresis (2-DE) master maps of control and treated A375 cells were generated by analysis with PDQuest software. Comparison between these maps showed up- and downregulation of 21 proteins, seven of which were upregulated and 14 were downregulated. The proteomics studies described here identify some proteins that are involved in mitochondrial dysfunction and apoptosis-associated proteins, including heat shock protein 60, heat shock protein beta-1, ubiquinol cytochrome c reductase complex core protein 1, isocitrate dehydrogenase (NAD) subunit alpha (down-regulated), and prohibitin (up-regulated), in A375 melanoma cells exposed to sinulariolide. Sinulariolide-induced apoptosis is relevant to mitochondrial-mediated apoptosis via caspase-dependent pathways, elucidated by the loss of mitochondrial membrane potential, release of cytochrome c, and activation of Bax, Bad and caspase-3/-9, as well as suppression of p-Bad, Bcl-xL and Bcl-2. Taken together, our results show that sinulariolide-induced apoptosis might be related to activation of the caspase cascade and mitochondria dysfunction pathways. Our results suggest that sinulariolide merits further evaluation as a chemotherapeutic agent for human melanoma.

## 1. Introduction

Melanoma, the deadliest form of skin cancer, is a malignant tumor of cutaneous melanocytes. The incidence rates of melanoma continue to rapidly rise throughout the world [1]. According to an estimation of the American Cancer Society, invasive melanoma diagnoses were estimated to have reached 70,230 cases in 2011 in the United States [2]. Metastatic melanoma is mostly incurable in diagnosed patients, because melanoma does not respond to most systemic treatments [3,4,5]. For patients with malignant melanoma, surgery is the most essential treatment [6,7]. Other medical measures such as chemotherapy and cytokine therapy have also been investigated. However, dacarbazine, the most active single agent against melanoma, has a response rate of approximately 15%–25% [8,9]. Cytokine therapy using high-dose interleukin (IL)-2 has an overall response rate of 12.5%, but the response rate does not approach that of dacarbazine [10,11]. Hence, malignant melanoma is an urgent medical and therapeutic issue. It is important to find new drugs and develop therapies against this highly malignant tumor.

Recently, many studies have analyzed marine biologically active compounds to discover new therapeutic drugs for the prevention or inhibition of cancer development [12,13,14,15,16,17]. Sinulariolide, an active compound isolated from the cultured soft coral *Sinularia flexibilis*, has been shown to have various biological properties, including anti-microbial and anti-bladder cancer activities [18,19]. In the current investigation, the effects of sinulariolide on melanoma cells were evaluated in terms of cell viability, cell migration, and flow cytometric analysis. The results indicated anti-tumor and apoptosis-induced effects of sinulariolide on A375 melanoma cells. A comparative proteomics analysis was performed to investigate the effects of sinulariolide on A375 melanoma cells. Several differential proteins identified after sinulariolide treatment by liquid chromatography-tandem mass spectrometry (LC-MS/MS) analysis were characterized as potential anti-cancer protein markers. Western blotting analysis was carried out to confirm the changes of protein expressions, which are associated with energy metabolism and cell apoptosis. Overall, these results could provide valuable information for drug development or potential strategies against human melanoma.

## 2. Results

### 2.1. The Cytotoxicity and Anti-Migratory Effects of Sinulariolide on A375 Cells

**Figure 1:**
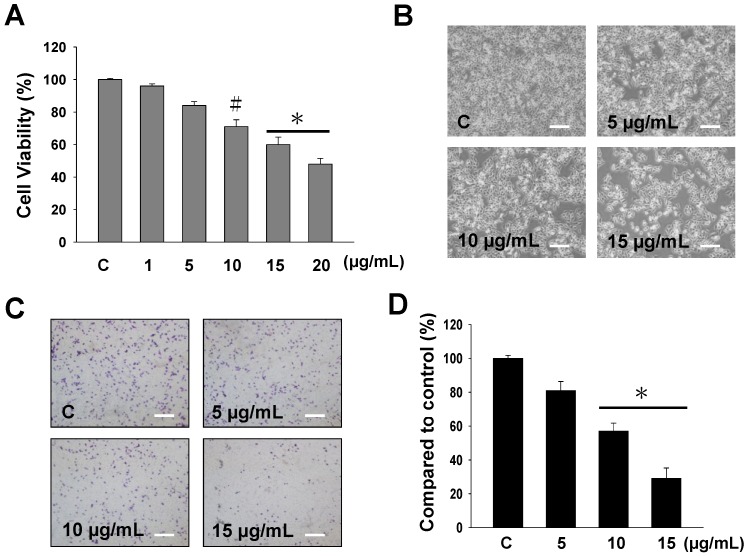
Evaluation of the cell cytoxicity and anti-migratory effects of sinulariolide on A375 melanoma cells. (**A**) The cell viability of A375 melanoma cells was found to be concentration-dependently suppressed by methylthiazoletetrazolium (MTT) assay (* *p* < 0.001, ^#^ *p* < 0.05); (**B**) Reduced cell population and morphological changes of A375 melanoma cells treated with different concentrations of sinulariolide (5, 10 and 15 μg/mL); (**C** and **D**) Sinulariolide from 5 to 15 μg/mL concentration-dependently decreased A375 cell migration (* *p* < 0.001). C: control, DMSO-treated cells. Scale bar (**B**,**C**) = 20 μm.

The potential cytotoxic effects of sinulariolide on human melanoma A375 cells were examined by methylthiazoletetrazolium (MTT) assay, morphological changes and cell migration assay. Figure 1A displays the cytotoxic effects of various concentrations of sinulariolide (1, 5, 10, 15, 20 μg/mL) on A375 melanoma cells. The results of the MTT assay revealed that sinulariolide concentration-dependently inhibited the proliferation of A375 melanoma cells (* *p* < 0.001, ^#^ *p* < 0.05) (Figure 1A). Morphological changes of sinulariolide-treated cells were observed by inverted light microscopy. The cell population of A375 cells obviously decreased after treatment with 10 and 15 μg/mL sinulariolide (Figure 1B). Data from the cell migration assay showed that 5–15 μg/mL sinulariolide suppressed A375 melanoma cell migration in a concentration-dependent manner, with suppression rates of approximately 21%, 42% and 72% for 5, 10 and 15 μg/mL sinulariolide treatment, respectively (Figure 1C,D).

### 2.2. Apoptosis Was Triggered by Sinulariolide in A375 Cells

**Figure 2:**
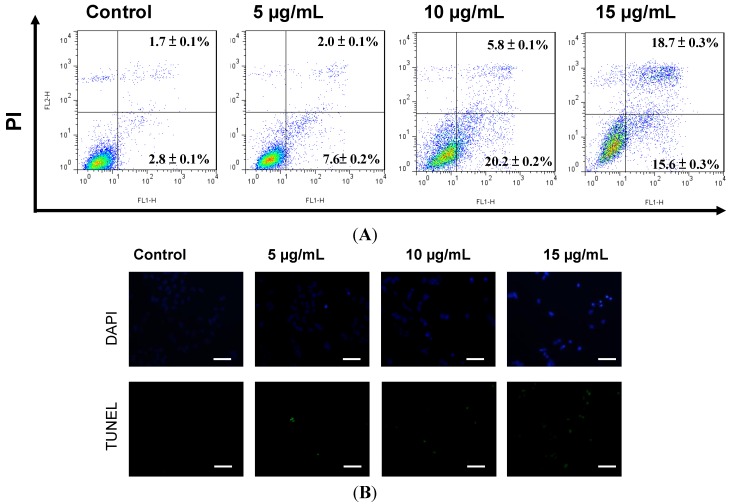
Sinulariolide-induced cell apoptosis of A375 melanoma cells. (**A**) Detection of apoptotic A375 cells after sinulariolide treatment by annexin V-fluoresceinisothiocyanate (FITC)/porpidium iodide (PI) analysis. Sinulariolide elevated early and late apoptosis in a concentration-dependent manner; (**B**) Detection of apoptotic cells by 4′,6-diamidino-2-phenyl iodide (DAPI) and terminal deoxynucleotidyl transferase dUTP nick end labeling (TUNEL) staining assay. A375 cells were treated with DMSO or sinulariolide at final concentrations of 5, 10 and 15 μg/mL for 24 h. Cells were harvested for DAPI and TUNEL staining as described in Materials and Methods. Scale bar = 50 μm.

It has previously been shown that sinulariolide induced apoptosis of TSGH cells [18]. A375 cells showed growth inhibition with sinulariolide treatment in a concentration-dependent manner. To investigate whether sinulariolide also induced apoptosis in melanoma cells, A375 cells were exposed to sinulariolide and were analyzed by flow cytometry-based annexin V-fluoresceinisothiocyanate (FITC)/porpidium iodide (PI) double staining using a flow cytometer. After treatment with control, 5, 10 and 15 μg/mL of sinulariolide, the percentages of cells displaying early apoptosis/late apoptosis were 2.8%/1.7%, 7.6%/2.0%, 20.2%/5.8% and 15.6%/18.7%, respectively (Figure 2A). These data showed that sinulariolide efficiently induced apoptosis of A375 cells. To further validate the sinulariolide-induced apoptosis of A375 cells, 4′,6-diamidino-2-phenyl iodide (DAPI) and Terminal deoxynucleotidyl transferase dUTP nick end labeling (TUNEL) stained assays were performed to evaluate the apoptosis. The results showed that massive apoptotic bodies were induced in A375 cells treated with 15 μg/mL of sinulariolide (Figure 2B). Altogether, these results demonstrated that treatment with sinulariolide is able to induce both early and late apoptosis in A375 melanoma cells.

### 2.3. Proteomic Analysis of A375 Cells Treated with Sinulariolide

**Figure 3:**
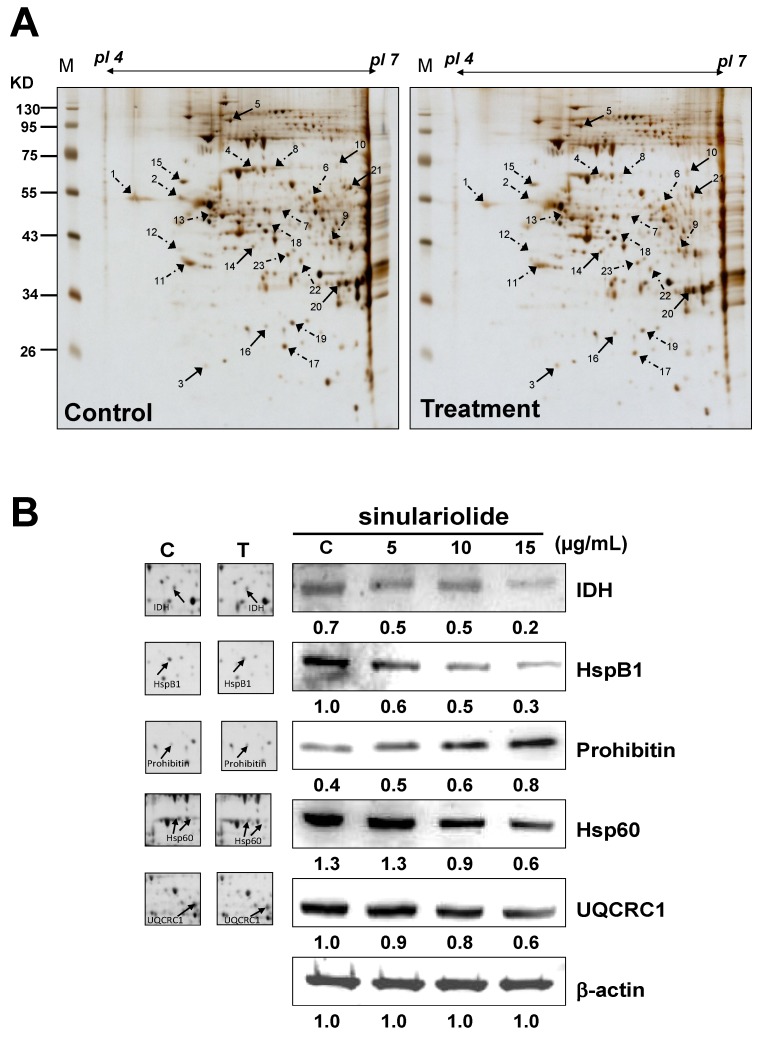
(**A**) Protein spots marked on the maps were considered differentially expressed as identified by liquid chromatography-tandem mass spectrometry (LC-MS/MS). The results were repeated three times; (**B**) Western blotting assay to validate the selected proteins identified by 2-DE, including isocitrate dehydrogenase (NAD) subunit alpha (IDH), heat shock protein beta-1 (HspB1), prohibitin, Hsp60, and ubiquinol cytochrome c reductase complex core protein 1 (UQCRC1). T: treatment; C: control, DMSO-treated cells. β-actin was used as the internal control.

Proteomic analysis was used for the identification of differentially expressed proteins between control and sinulariolide-treated A375 cells. A375 cells were treated with 15 μg/mL sinulariolide for 24 h and then harvested. Total proteins were extracted from cultured cells with lysis buffer and subjected to two-dimensional gel electrophoresis (2-DE). A total of 100 μg protein was dissected with 2-DE (*p*I 4–7) and visualized by silver staining (Figure 3A). PDQuest image analysis software (Bio-Rad, Hercules, CA, USA) was employed for the detection of differential protein spots, which were defined as proteins showing a more than one and a half-fold intensity difference in 2-DE maps between the treated A375 cells and the control samples. Protein identification was carried out by LC-MS/MS analysis after gel digestion. MASCOT protein identification search software was used for the identification of the differential protein spots. A total of 21 differential protein spots were successfully identified. A list of the identified proteins with their MASCOT scores, theoretical MW/*p*I, tandem mass spectrometry (MS/MS) matched sequences, coverage, and fold changes in expression levels (upregulation or downregulation) is given in Table 1. There were seven differential proteins upregulated after sinulariolide treatment: Rho GDP-dissociation inhibitor 1, transitional endoplasmic reticulum ATPase, Lamin-A/C, stomatin-like protein 2, prohibitin, translation initiation factor eIF-2B subunit alpha and retinal dehydrogenase 2. A total of 14 differential proteins were downregulated after sinulariolide treatment: Calreticulin, vimentin, 60 kDa heat shock protein (Hsp60), ubiquinol cytochrome c reductase complex core protein 1 (UQCRC1), glutathione synthetase, DnaJ homolog subfamily B member 11, nucleophosmin, reticulocalbin-1, protein disulfide-isomerase, platelet-activating factor acetylhydrolase IB subunit beta, heterogeneous nuclear ribonucleoprotein F (hnRNP F), heat shock protein beta-1 (HspB1), isocitrate dehydrogenase (NAD) subunit alpha (IDH) and pyruvate dehydrogenase E1 component subunit beta. Differential proteins such as UQCRC1, IDH, prohibitin, Hsp60 and HspB1 are associated with anti-proliferation and induction of apoptosis, and the changes in these proteins were verified by western blot. Altogether, the differentially expressed proteins validated by western blot analysis were in agreement with the 2-DE data (Figure 3B).

**Table 1:** Protein identification by LC/MS/MS.

Spot No.	Protein name	Accession No.	Calculated Mw/*p*I	Peptide matched	Sequence covered %	MASCOT score	Regulation (fold change) *
1	Calreticulin precursor	P27797	48.11/4.29	35	40	251	−1.6
2	Vimentin	P08670	53.61/5.06	26	39	272	−2.2
3	Rho GDP-dissociation inhibitor 1 (Rho-GDI)	P52565	23.19/5.02	5	26	68	+2.3
4	60 kDa heat shock protein (Hsp60)	P10809	61.01/5.7	51	47	545	−2.6
5	Transitional endoplasmic reticulum ATPase	P55072	89.26/5.14	49	47	439	+1.8
6	Ubiquinol cytochrome c reductase complex core protein 1 (UQCRC1)	P31930	52.61/5.94	5	10	53	−1.9
7	Glutathione synthetase	P48637	52.35/5.67	5	10	123	−2.8
8	60 kDa heat shock protein (Hsp60)	P10809	61.01/5.7	3	8	66	−2.5
9	DnaJ homolog subfamily B member 11 precursor	Q9UBS4	40.48/5.81	3	9	40	−2.2
10	Lamin-A/C (70 kDa lamin)	P02545	74.09/6.57	3	10	71	+1.8
11	Nucleophosmin (NPM)	P06748	32.55/4.64	28	39	216	−1.9
12	Reticulocalbin-1 precursor	Q15293	38.86/4.86	4	9	84	−2.4
13	Vimentin	P08670	53.61/5.06	7	8	101	−1.7
14	Stomatin-like protein 2	Q9UJZ1	38.51/6.88	29	46	331	+2.0
15	Protein disulfide-isomerase precursor (PDI)	P07237	57.08/4.76	23	35	205	−2.1
16	Prohibitin	P35232	29.78/5.57	45	67	660	+1.8
17	Platelet-activating factor acetylhydrolase IB subunit beta	P68402	25.55/5.57	4	9	46	−2.2
18	Heterogeneous nuclear ribonucleoprotein F	P52597	45.64/5.38	8	21	121	−1.8
19	Heat shock protein beta-1 (HspB1)	P04792	22.76/5.98	9	15	102	−2.6
20	Translation initiation factor eIF-2B subunit alpha	Q14232	33.69/6.9	1	4	47	+2.5
21	Retinal dehydrogenase 2	O94788	56.68/5.79	3	2	54	+2.3
22	Isocitrate dehydrogenase (NAD) subunit alpha (IDH)	P50213	39.56/6.47	10	17	148	−1.9
23	Pyruvate dehydrogenase E1 component subunit beta	P11177	39.19/6.2	3	10	68	−1.5

* Regulation (fold change) of differentially-expressed proteins was calculated after 24 h of sinulariolide treatment.

### 2.4. Sinulariolide Induced Mitochondrial Depolarization and Activated the Mitochondrial-Related Pathway, Resulting in Cell Apoptosis

**Figure 4:**
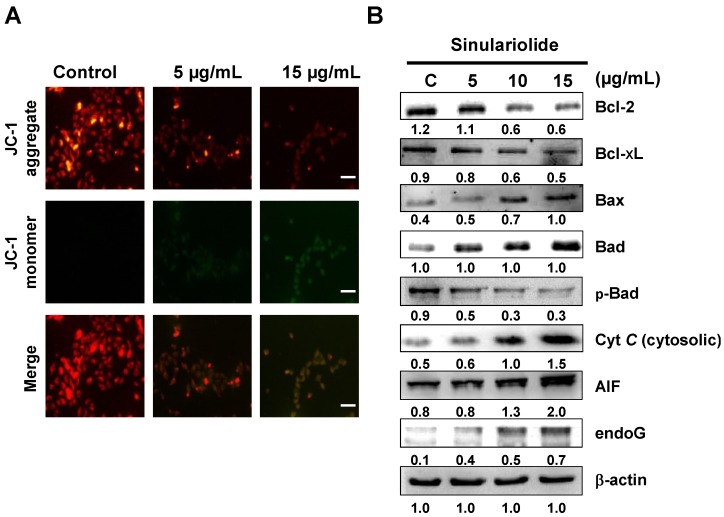
Sinulariolide induced apoptosis via mitochondria potential (ΔΨm) change and the mitochondria-related pathway in A375 melanoma cells. (**A**) A375 melanoma cells were treated with or without sinulariolide. The mitochondria potential change in melanoma cells was detected by staining with JC-1 and was analyzed by fluorescence microscopy; (**B**) Western blotting data showed the changes of Bcl-2, Bcl-xL, Bax, Bad, p-Bad, cytosolic cytochrome c, AIF, and endo G expression in melanoma cells treated with different concentrations of sinulariolide. Scale bar = 50 μm. β-actin was used as the internal control.

From the 2-DE results, some mitochondrial-related proteins were identified upon sinulariolide treatment, such as IDH and UQCRC1, which are involved in energy production. Therefore, we measured the loss of mitochondrial membrane potential (ΔΨm) induced by sinulariolide using JC-1 dye. Fluorescence microscopy showed that untreated melanoma cells had strong red fluorescence (J-aggregation) and weak green florescence (JC-1 monomer). However, many 15 μg/mL sinulariolide-treated A375 cells showed a significant reduction in red fluorescence and increased signals of green fluorescence due to loss of ΔΨm following treatment with sinulariolide (Figure 4A). The mitochondrial-related apoptosis pathway plays an important role in apoptosis. To verify this mitochondrial-related apoptosis pathway, we further analyzed several mitochondrial-related apoptotic markers, such as Bax, Bad, p-Bad, cytosolic cytochrome c, endo G, apoptosis-inducing factor (AIF), Bcl-2, and Bcl-xL. The results are displayed in Figure 4B, and show that sinulariolide significantly increases the expression levels of Bax, Bad, AIF, endo G and cytosolic cytochrome c in A375 cells. In contrast, the expression levels of Bcl-2, Bcl-xL, and p-Bad decrease after sinulariolide treatment.

### 2.5. Sinulariolide Activates the Caspase-Dependent Pathway, Leading to Cell Apoptosis

**Figure 5:**
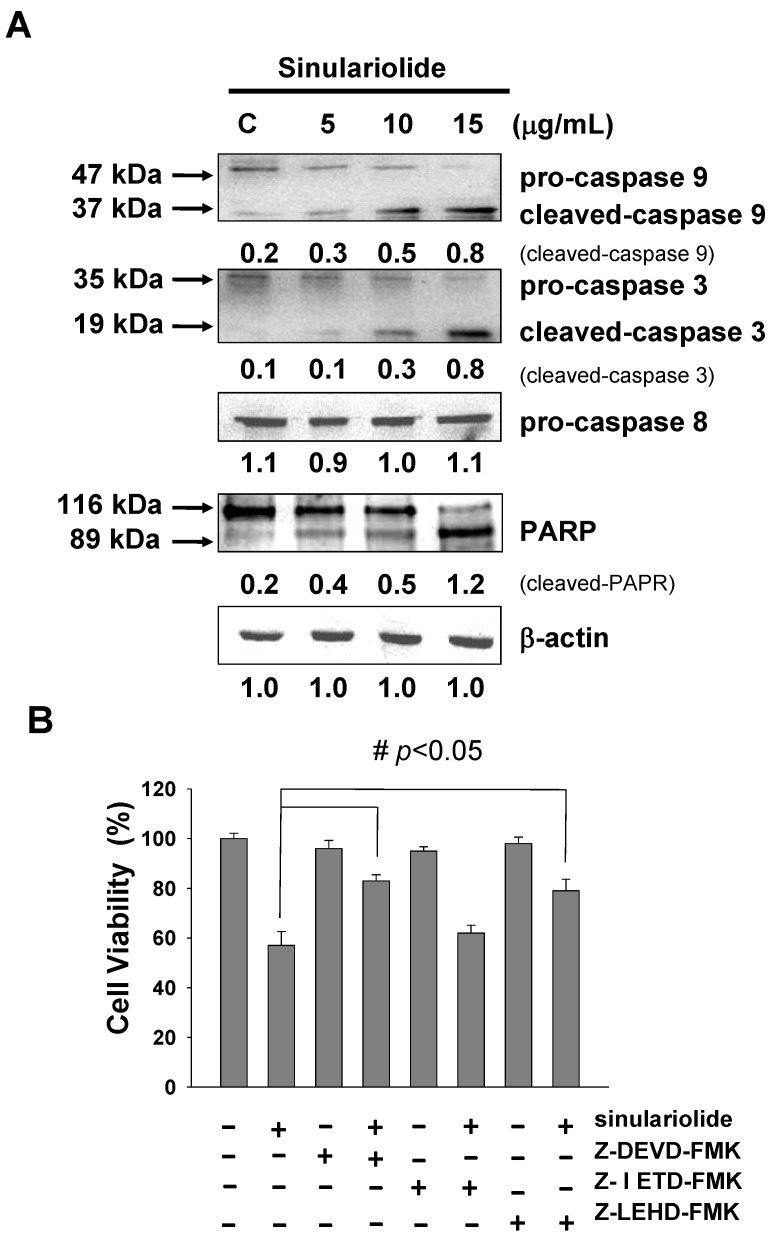
Sinulariolide induced apoptosis through the caspase-dependent pathway. (**A**) Representative western blots showing changes in the apoptosis-associated protein expression levels after treatment with sinulariolide in A375 melanoma cells; (**B**) Caspase-3, -8, and -9 inhibitors affected the A375 melanoma cell viability after sinulariolide treatment. Cells were seeded on to a 24-well plate and pretreated with or without Z-DEVD-FMK, Z-IETD-FMK and Z-LEHD-FMK, followed by treatment with 15 μg/mL sinulariolide. The cells were then harvested at 24 h and subjected to MTT assay for the evaluation of cell viability. ^#^ *p* < 0.05 as compared to the control groups.

Differential expression levels of caspase-3, caspase-8 and caspase-9 have been reported to be involved in the cell apoptosis pathway. In Figure 5A, western blotting data showed that the expression levels of both pro-caspase-3 and pro-caspase-9 were decreased in the sinulariolide-treated cells. The expression levels of cleaved-caspase-3 (17 kDa proteolytic fragments) and cleaved-caspased-9 (37 kDa proteolytic fragments) were elevated after sinulariolide treatment. The expression level of caspase-8 did not change. Similarly, cleaved-poly ADP ribose polymerase (PARP) (89 kDa proteolytic fragments) also increased in expression upon sinulariolide treatment (Figure 5A). These results showed that the caspase-dependent pathway was activated upon sinulariolide treatment. We examined whether caspase activation was involved in sinulariolide-induced cell apoptosis. Three caspase inhibitors, Z-DEVD-FMK (caspase-3 inhibitor), Z-IETD-FMK (caspase-8 inhibitor), and Z-LEHD-FMK (caspase-9 inhibitor), were used to examine whether they were able to block sinulariolide-induced cell apoptosis. Only Z-DEVD-FMK and Z-LEHD-FMK inhibited the sinulariolide-induced cell apoptosis (Figure 5B). These data suggest that caspase-3 and caspase-9 but not caspase-8 are involved in melanoma cell apoptosis after sinulariolide treatment.

## 3. Discussion

In the current study, sinulariolide, isolated from the cultured soft coral *Sinularia flexibilis*, exerted cell cytotoxic and apoptosis-inducing effects on A375 melanoma cells. Our study examined the effects of sinulariolide on the cell viability. Morphological changes and migration demonstrated a concentration-dependent effect (Figure 1). The induction of apoptosis was determined by annexin V-FITC/PI staining using a flow cytometer. Treatment with sinulariolide concentration-dependently increased the early and late apoptotic rates in A375 melanoma cells (Figure 2). These results demonstrate that sinulariolide exerts anti-proliferation and apoptosis-inducing activities against melanoma cells.

The proteomic data could provide clues for the investigation of potential markers and apoptosis-inducing effects of sinulariolide on A375 melanoma cells at the molecular level. Proteins related to the effects of sinulariolide were identified by comparative proteomic analysis. We found that changes in some crucial proteins, such as Hsp60, HspB1, prohibitin, IDH, and UQCRC1, were associated with apoptosis or the mitochondrial function against melanoma cells. Hsp60 siRNA induces mitochondrial dysfunction and activates both p53-dependent and mitochondrial apoptosis [20]. HspB1, also known as heat shock protein 27, has been indicated to be a critical marker in several cancer cells [21]. Hsp B1 (Hsp27) siRNA leads to downregulation of Bcl-2 and upregulation of Bax [22]. Our previous work has shown downregulation of Hsp60 in BFTC bladder cancer cells treated with 13-acetoxysarcocrasslide and CAL-27 oral squamous cell carcinoma treated with 11-dehydrosinulariolide, which exerts anti-tumor effects *in vitro* [13,23]. Recent results have shown that treatment with sinulariolide significantly enhances apoptosis in A375 melanoma cells and reduces the Hsp60 and Hsp27 expression levels. Prohibitin, which is located in mitochondria, was found to regulate apoptosis, cellular signaling, cell migration, and cell proliferation, and stabilize mitochondria proteins [24,25,26,27]. The translocation of prohibitin to mitochondria couples with simultaneous translocation of p53, which is highly correlated with inhibition of cancer growth. Up-regulation of IDH has been reported to correlate with the metastasis of human breast cancer [28]. Inhibition of IDH resulted in curcumin-induced apoptosis in HCT116 colon cancer cells [29]. UQCRC1 is a key subunit of complex III of the mitochondrial respiratory chain. Up-regulation of UQCRC1 might contribute to the rapid proliferation of cancer cells by increased synthesis of ATP. Some anti-cancer drugs can inhibit the mitochondrial respiratory chain at complex III [30]. This suggests that the anti-cancer effects of sinulariolide may be correlated with mitochondrial function in A375 cells and associated with the reduction of IDH and UQCRC1 and enhancement of prohibitin.

The apoptosis-induced activity is a good basis for anti-cancer therapies and also a valuable guide to predict tumor response after anticancer administered treatment. The specific induction of human cancer cell apoptosis using chemotherapy critically benefits anti-cancer drug development [12]. Apoptotic processes can be divided into two major pathways: One is mediated by the plasma membrane (extrinsic pathways) and one is mediated by cells (intrinsic pathways) [31]. The intrinsic pathways that initiate apoptosis involve a large number of intracellular signals that are located in the endoplasmic reticulum or mitochondria [32]. In the mitochondria-dependent apoptotic pathway, the suppression of anti-apoptotic proteins such as Bcl-xL, Bcl-2 and Mcl-1, and activation of pro-apoptotic Bax, Bid and Bak, have been reported to alter the mitochondrial membrane potential and release mitochondrial apoptotic factors [33].

Loss of mitochondrial membrane potential leads to the release of cytochrome c, AIF and endo G from mitochondria. AIF and endo G, which are known to induce caspase-independent apoptosis, might be released into the cytosol upon loss of mitochondrial membrane potential. Cytochrome c is released from mitochondrial inter-membrane spaces and then binds to ARAF1, leading to the activation of caspase-9 and further activating the downstream effector caspase-3. Activation of caspase-3 subsequently cleaves poly (ADP-ribose) polymerase (PARP), which is further activated [34]. Thus, sinulariolide induces apoptosis in A375 cells through the mitochondria intrinsic pathway. We found that sinulariolide treatment resulted in reduced levels of mitochondrial membrane potential and led to the release of cytochrome c, AIF and endo G from mitochondria (Figure 4). Furthermore, sinulariolide also suppressed anti-apoptotic factors Bcl-2, Bcl-xL and Mcl-1 but promoted pro-apoptotic factors Bad and Bax (Figure 4). Sinulariolide treatment resulted in concentration-dependent activation of caspase-3, caspase-9 and PARP (Figure 5). The present data support that sinulariolide can induce apoptosis of A375 cells through activation of the caspase cascade. 

Overall, our results indicate that sinulariolide increases apoptosis of A375 cells, which is associated with the intrinsic mitochondria dysfunction pathway and activation of the caspase cascade.

## 4. Materials and Methods

### 4.1. Materials

Immobilized pH gradient (IPG) buffer and isoelectric focusing (IEF) strips were purchased from GE Healthcare (Buckinghamshire, UK). Cell Extraction Buffer was obtained from BioSource International (Camarillo, CA, USA). Rabbit anti-human AIF, endo G and prohibitin antibodies were obtained from Epitomics (Burlingame, CA, USA). Rabbit anti-human Hsp60, HspB1, UQCRC1 and IDH antibodies were obtained from ProteinTech Group (Chicago, IL, USA). Rabbit anti-human pro-casapse-3, pro-caspase-9, pro-caspase-8, cleaved caspase-3, cleaved caspase-9, PARP, cytochrome c, Bax, Bad, p-Bad, Bcl-2 and Bcl-xL antibodies were obtained from Cell Signaling Technology (Danvers, MA, USA). Protease inhibitor cocktail, dimethyl sulfoxide (DMSO), Z-DEVD-FMK (caspase-3 inhibitor), Z-IETD-FMK (caspase-8 inhibitor), Z-LEHD-FMK (caspase-9 inhibitor), 3-(4,5-dimethylthiazol-2-yl)-2,5-diphenyltetrazolium bromide (MTT), and rabbit anti-human β-actin antibodies were obtained from Sigma (St. Louis, MO, USA). Polyvinylidene difluoride (PVDF) membranes and goat anti-rabbit and horseradish peroxidase conjugated IgG were obtained from Millipore (Bellerica, MA, USA). Chemiliminescent horseradish peroxidase (HRP) substrate was from Pierce (Rockford, IL, USA). A DAPI fluorescent kit was obtained from Promega (Madison, WI, USA). Sinulariolide was isolated from the cultured soft coral *Sinularia flexibilis* according to the reported procedures [35]*.* DMSO was used to dissolve sinulariolide.

### 4.2. Cell Culture

A375 melanoma cells were purchased from the Food Industry Research and Development Institute (Hsinchu, Taiwan) and were grown in DMEM (Biowest, Nuaillé, France), 4 mM l-glutamine, 10% (v/v) fetal bovine serum, 100 μg/mL streptomycin, 100 U/mL penicillin and 1 mM sodium pyruvate in a humidified atmosphere of 5% CO_2_ in air at 37 °C. 

### 4.3. Determination of Cell Viability

The cell viability effect of sinulariolide against A375 cells was examined by calorimetric tetrazolium (MTT) assay [14]. Briefly, A375 cells were seeded in 96-well plates at a density of 1 × 10^5^/cm^2^ in complete medium (with 10% fetal bovine serum) and treated with different concentrations of sinulariolide (1, 5, 10, 15 and 20 μg/mL) for 24 h. Cells treated with DMSO without sinulariolide were used as a blank control. After incubation, cells were washed and 50 μL MTT solution added (1 mg/mL in phosphate buffered saline (PBS) buffer) at 37 °C for 4 h. Then, cells were lysed with 200 μL DMSO. The absorbance was determined at 595 nm on a microtiter plate ELISA reader (Bio-Rad, Hercules, CA, USA) with DMSO as the blank. All experiments were carried out in triplicate to confirm the reproducibility, and the results of three independent experiments were used for statistical analysis.

### 4.4. Cell Migration Assay

The cell migration assay was performed according to the methods described by Su *et al.* [36]. A375 melanoma cells were seeded onto a Boyden chamber (Neuro Probe, Cabin John, MD, USA) at 10^4^ cells/well in serum-free media for 24 h. Melanoma cells with or without sinulariolide treatment were allowed to migrate for 24 h. The migrated cells on the lower site were fixed with 100% methanol and then stained with 5% Giemsa (Merck, Germany). Cell numbers were observed and counted using a 100**×** light microscope.

### 4.5. Quantitative Detection of Apoptosis by Flow Cytometry

To determine the apoptosis induced by sinulariolide in A375 melanoma cells, an annexin V-FITC Apoptosis Detection kit (Pharmingen, San Diego, CA, USA) was used and the method was as according to a previous study [37]. A total of 1 × 10^6^ cells were seeded onto a 5-cm Petri dish and treated with or without sinulariolide for 24 h, and subsequently cells were stained with annexin V-FITC and propidium iodide (PI) for 30 min at 37 °C according to the manufacturer’s protocol. Apoptotic cells were then assessed using a FACScan flow cytometer and Cell-Quest software (Becton-Dickinson, Mansfield, MA, USA).

### 4.6. Protein Extraction and Concentration Estimation

A375 melanoma cells were treated without or with various concentrations of sinulariolide (0, 5, 10 and 15 μg/mL) for 24 h and then lysed with Cell Extraction Buffer (BioSource). The total protein in the supernatant was then precipitated out by triple the volume of 10% trichloroacetic acid (TCA)/acetone solution overnight at −20 °C. The precipitated proteins were suspended in a rehydration buffer (6 M urea, 2 M thiourea, 0.5% IPG buffer, 0.5% 3-[(3-cholamidopropyl)dimethylammonio]-1-propanesulfonate (CHAPS), 20 mM dithiothreitol (DTT), and 0.002% bromophenol blue) at 4 °C overnight. The protein concentrations were determined using a 2-D Quant Kit (GE Healthcare).

### 4.7. Two-Dimensional Gel Electrophoresis, Image Analysis and Protein Identification by LC-MS/MS

Two-dimensional gel electrophoresis (2-DE), image analysis and LC-MS/MS analysis were carried out by methods similar to those used in our previous study [13,23,38]. For 2-DE, protein samples (100 μg) from control cells and cells treated with 15 μg/mL sinulariolide were applied for first dimension electrophoresis (isoelectric focusing) using a GE Healthcare Ettan IPGphor 3. Then, the equilibrated strip was loaded onto the top of a sodium dodecyl sulfate polyacrylamide gel electrophoresis (SDS-PAGE gel) (12.5%) for second dimension electrophoresis using an SE 600 Ruby (Hoeffer, Holliston, MA, USA). 2-DE images were obtained in triplicate for each sample and normalization prior to statistical analysis was performed to ensure reproducibility. The 2-DE gels were stained by silver. The silver-stained gels were then scanned and analyzed using PDQuest image analysis software (Bio-Rad). Protein spots showing more than a one and a half-fold intensity difference were considered statistically significant in 2-DE between the sinulariolide treated A375 cells and the control. The protein spots of interest were excised from the 2-DE gels. After digestion with trypsin, the excised protein spots were used for identification by LC-MS/MS analysis using an AB SCIEX QTRAP^®^ 5500Q mass spectrometer (Applied Biosystems, Framingham, CA, USA). For the LC-MS/MS procedure, the peptide mixture was separated by a reversed phase column (Agilent Zobax 2.1 mm × 150 mm C18 column, Santa Clara, CA, USA) on a nano LC system (Agilent NanoLC 1200 System). The LC-MS/MS analysis employed a 10-mm online trapping and desalting step followed by a linear gradient from 5% to 60% acetonitrile containing 0.1% formic acid over 60 min. The scan range was from *m*/*z* 100 to1000 for MS. The raw data were processed into the text file format of WIFF using Analyst 1.5.1. All MS/MS spectra of identified peptides were further verified by manual interpretation.

## 4.8. DAPI and TUNEL Stain

A375 melanoma cells (1 × 10^5^ cells/well) in a 12-well plate were treated with 5, 10, 15 μg/mL sinulariolide for 24 h, and DMSO was added as the control. Cells in each treatment and control group were fixed with 4% paraformaldehyde in PBS solution for 15 min and stained by DAPI according to the manufacturer’s instructions. The DeadEnd™ Fluorometric TUNEL System (Promega) was used to detect nuclear DNA fragmentation according to the manufacturer’s manual. The cells were photographed under a fluorescence microscope (Olympus IX71 CTS, Chinetek Scientific, Hong Kong, China).

## 4.9. Mitochondria and Cytosol Fractionation

The mitochondria and cytosol fractions were separated using a mitochondria/cytosol fractionation kit (BioSource) by the methods described in our previous report [18].

## 4.10. Assessment of Mitochondrial Membrane Potential (ΔΨm)

The mitochondrial membrane potential (ΔΨm) was examined by fluorescence microscopy following staining with the cationic dye JC-1 (Biotium, Hayward, CA, USA). A375 cells (1 × 10^5^ cells/well) in a 12-well plate were treated with different concentrations of sinulariolide (0, 5 and 15 μg/mL) and grown in 5% CO_2_ at 37 °C. After incubation, cells were harvested and washed twice with PBS, then 70 μL of JC-1 staining solution were added and incubation performed at 37 °C in the dark for 30 min. After a brief wash with serum-free medium, cells were directly observed by fluorescence microscopy (Olympus IX71 CTS, Chinetek Scientific, Hong Kong, China).

## 4.11. Western Blot Analysis

The treated samples and control samples (25 μg) were separated by 12.5% SDS-PAGE, and then transferred to a PVDF membrane (Millipore) for 1.5 h at 400 mA using Transphor TE 62 (Hoeffer). The membranes were incubated with 5% dehydrated skim milk to block nonspecific protein binding, and then incubated with primary antibodies at 4 °C for overnight. The primary antibodies were anti-human prohibitin, IDH, UQCRC1, Hsp60, HspB1, PARP, pro-caspase-3, cleaved-caspase-3, pro-caspase-9, cleaved-caspase-9, pro-caspase-8, Cyt c, Bax, Bad, p-Bad, Bcl-2, Bxl-xL, AIF, endo G and β-actin antibodies. The secondary antibodies (horseradish peroxidase conjugated goat anti-rabbit, 1:5000 in blocking solution) were added and incubated for 2 h and then visualized using chemiluminesence (Pierce Biotechnology, Rockford, IL, USA). The western blot data were quantified using Image J software.

## 4.12. Statistical Analysis

Data for the MTT assay, cell migration assay and flow cytometric analysis were pooled from three independent experiments and are expressed as the mean ± standard error of mean (SEM). Data analysis of variance (ANOVA) by the Tukey-Kramer test was performed using GraphPad InStat 3 (San Diego, CA, USA) to determine the significant differences (*p* ≤ 0.05) as compared with the experimental groups [14].

## 5. Conclusions

**Figure 6:**
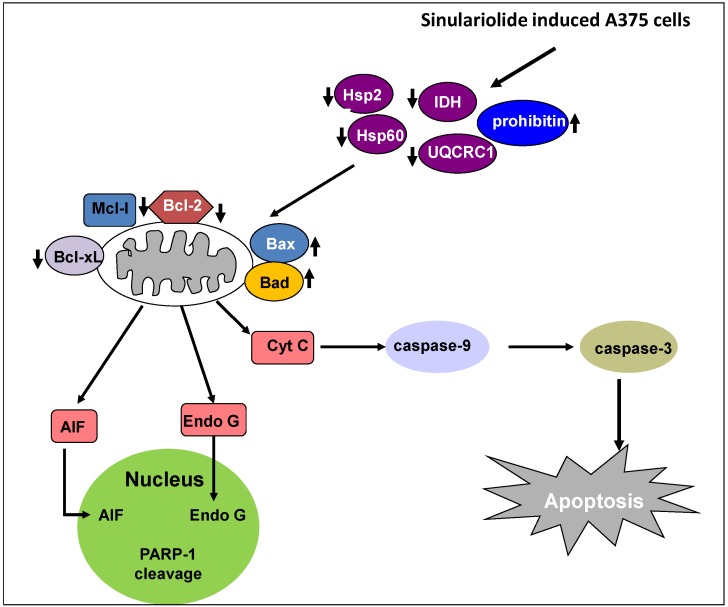
Sinulariolide induces A375 melanoma cell apoptosis through the mitochondrial-related apoptosis pathway and activation of the caspase cascade.

In conclusion, our results establish that the natural marine compound sinulariolide possesses anti-cancer activity through the induction of cellular apoptosis in A375 melanoma cells. Proteomic analysis of the differentially expressed proteins profiled indicated that energy metabolism proteins were related to the apoptotic progress. Furthermore, our findings indicate that sinulariolide performs anti-cancer activity involving induction of cellular apoptosis. Overall, this study indicates that sinulariolide-induced apoptosis can be summarized as shown in Figure 6. Sinulariolide-induced apoptosis might be related to the activation of the caspase cascade and the mitochondrial-related apoptosis pathway. Further *in vivo* evaluation of the anti-melanoma cancer activity of sinulariolide in an animal model is needed in the future. The findings of our study also benefit melanoma pharmaceutical development in the clinical setting.
